# PD215 Assessing Drug Pricing Disparities In Brazil’s Public And Private Healthcare Sectors

**DOI:** 10.1017/S0266462324004367

**Published:** 2025-01-07

**Authors:** Marcus Carvalho Borin, Carina Rejane Martins, Daniel Pitchon dos Reis, Geraldo Jose Coelho Ribeiro, Julia Teixeira Tupinambas, Karina de Castro Zocrato, Lelia Maria de Almeida Carvalho, Marcela Pinto de Freitas, Maria da Gloria Cruvinel Horta, Mariana Michel Barbosa, Mariza Cristina Torres Talim, Sergio Adriano Loureiro Bersan, Silvana Marcia Bruschi Kelles

## Abstract

**Introduction:**

In Brazil, equitable access to medications is critical. There are significant pricing disparities between the National Health System and private health care, which are influenced by the National Committee for Health Technology Incorporation (CONITEC) and Law 14.307. This study investigated these disparities, with aim of proposing strategies for equitable access and sustainability in health care.

**Methods:**

This analysis compared prices between the public and private sectors for trastuzumab and adalimumab. Public sector prices were obtained from the Health Prices Database (HPD) and private sector prices were obtained from the Unimed National Table of Materials and Medications (TNUMM), as of May 2023. The study evaluated the extent of pricing discrepancies, considering Drug Market Regulation Chamber ceiling prices and industry discounts.

**Results:**

The cost of the trastuzumab biosimilar, KANJINTI® (Amgen Inc.), was BRL15.79 (USD3.24) per mg in the private sector, compared with BRL4.50 (USD0.92) per mg in the public sector (a 250% difference). The original version of adalimumab, HUMIRA® (AbbVie), was priced at BRL5,450.38 (USD1,120.53) in the TNUMM versus BRL2,445.46 (USD502.33) in the HPD (a 123% difference). The adalimumab biosimilar, HYRIMOZ® (Sandoz Inc.), was priced at BRL7,723.99 (USD1,586.87) in the TNUMM compared with BRL2,449.19 (USD503.05) in the HPD (a 215% price discrepancy).

**Conclusions:**

The study highlights significant disparities in drug pricing between Brazil’s public and private healthcare sectors. These disparities affect the financial sustainability of private health entities and elevate costs for consumers, potentially increasing reliance on the National Health System. Policy revisions, price parity strategies, and further studies are vital for a sustainable healthcare system.

